# Colonization and Healthcare-Associated Infection of Carbapenem-Resistant *Enterobacteriaceae*, Data from Polish Hospital with High Incidence of Carbapenem-Resistant *Enterobacteriaceae,* Does Active Target Screening Matter?

**DOI:** 10.3390/microorganisms11020437

**Published:** 2023-02-09

**Authors:** Iwona Pawłowska, Grzegorz Ziółkowski, Estera Jachowicz-Matczak, Michał Stasiowski, Mateusz Gajda, Jadwiga Wójkowska-Mach

**Affiliations:** 1 St. Barbara Specialized Regional Hospital No. 5, Division of Microbiology and Epidemiology, 41-200 Sosnowiec, Poland; ivi5@op.pl; 2 Prof. Kornel Gibiński University Clinical Center, Medical University of Silesia, ul. Medyków 14, 40-572 Katowice, Poland; nc3@wp.pl; 3 Department of Microbiology, Faculty of Medicine, Jagiellonian University Medical College, 31-121 Krakow, Poland; estera.jachowicz-matczak@uj.edu.pl (E.J.-M.); mateusz14.gajda@uj.edu.pl (M.G.); 4 Chair and Department of Emergency Medicine, Faculty of Medical Sciences in Zabrze, Medical University of Silesia, 40-055 Katowice, Poland; mstasiowski.anest@gmail.com; 5 Department of Anaesthesiology and Intensive Therapy, St. Barbara’s Specialized Regional Hospital in Sosnowiec, pl. Medyków 1, 41-200 Sosnowiec, Poland

**Keywords:** carbapenemase-producing *Enterobacterales*, *K. pneumoniae*, resistance

## Abstract

The objective of the study was to analyse the incidence of carbapenem-resistant *Enterobacteriaceae* (CRE) at a provincial hospital from 2019–2021. Multiplex PCR was used to detect the presence of carbapenemase genes. There were 399 cases of CRE detected in total in the analysed period, including 104 healthcare-associated infections. Out of the isolated CRE, 97.7% were *Klebsiella pneumoniae* with OXA-48 or KPC genes. Overall, among the identified CRE genes, the most frequently present genes were the ones mediating oxacillinase OXA-48 (71%) and KPC (26%), and significantly less often New Delhi NDM metallo-β-lactamase (2.5%). Moreover, two isolates produced two carbapenemases, i.e., OXA-48 and KPC. The conducted research demonstrates that there is a constant need for continuous monitoring of the occurrence of CRE strains and the hospital antibiotic policy, as well as the implementation of procedures to prevent CRE transmission by medical personnel and hospital support staff.

## 1. Introduction

Carbapenems are bactericidal β-lactam antibiotics with a broad antibacterial spectrum and wide clinical use. They include imipenem, meropenem, ertapenem, and doripenem, and their antimicrobial activity may vary [1]. They are among the newest groups of antibiotics employed in medicine. The first compound isolated from this group was thienamycin produced by *Streptomyces cattleya*, however, the process of obtaining and purifying the antibiotic proved troublesome. The real revolution was the discovery of a synthetic derivative of thienamycin, imipenem. However, in spite of the relatively short history of their application worldwide, there is a trend of rapidly increasing resistance to carbapenems, which is all the more worrisome given that these drugs are often the so-called ‘last resort’ [2]. The rapid and global expansion of carbapenemase-producing strains, including CRE (carbapenem-resistant *Enterobacteriaceae*), severely limited the options for antibiotic treatment of healthcare-associated infections, particularly in the past two decades [3]. Unfortunately, the COVID-19 pandemic was also conducive to the emergence of extremely resistant microbes and the increased prevalence of carbapenem resistance, which probably results from the increased and uncontrolled use of broad-spectrum antibiotics in COVID-19 patients [4,5]. Some countries noted a significant increase in the consumption of antibiotics in the early pandemic period, e.g., in United Kingdom [6]. CRE are among the top three multidrug-resistant pathogens on the WHO priority list [7], therefore, their appearance in the hospital should lead to the implementation of active epidemiological surveillance and changes in the hospital antibiotic policy aimed at reducing the scale of the overuse of antimicrobial agents [8]. 

The most significant mechanism causing carbapenem resistance in *Enterobacteriaceae* is the production of carbapenemases. Three major classes of carbapenemases have been largely associated with the global spread of CRE: *Klebsiella pneumoniae* carbapenemase KPC (Ambler class A), metallo-β-lactamases (MβL) (Ambler class B, e.g., NDM, VIM, and IMP), and OXA-48 (Ambler class D) [9]. The problem of carbapenem-resistant *Enterobacteriaceae* is more intense in some countries with a high prevalence of CRE, such as Greece [10].

One of the vital elements of the surveillance of infections, especially CRE healthcare-associated infections (HAIs), is CRE screening for effective control of CRE spreading and transmission-based precautions. The aim of this study was to analyse and explore the dependency of colonization and infection caused by CRE bacilli in a Polish provincial hospital in a 3-year active routine target surveillance based on real-time PCR-CRE identification of five of the most widespread genes from the carbapenemase family, NDM, KPC, OXA-48, VIM, and IMP. 

## 2. Materials and Methods

The analysis included patients hospitalized in the period from 2019–2021 at St. Barbara Specialized Regional Hospital No. 5 in Sosnowiec, Poland. It is the biggest hospital complex in southern Poland, with about 30,000 annual hospitalizations. The structure of the hospital comprises 5 clinical departments, including the Clinical Department of Anaesthesiology and Intensive Care. This entity is the only Multi-organ Trauma Centre in the Silesian Voivodship, which offers treatment for patients with severe and complicated diseases. 

The analysis encompassed patients with confirmed colonization and/or infection with CR-*Enterobacteriaceae* strains. Infection was defined as an instance of isolation of a CRE strain from clinically significant material, with simultaneous occurrence of clinical signs of infection caused by the microorganism isolated. Material that was considered clinically significant was as follows: blood and bodily fluids, i.e., cerebrospinal, synovial, pleural fluids, wound material, material collected intraoperatively, bronchial lavage, and urine. Colonization was defined as isolation of the CRE strain from rectal or perirectal swab from patient without clinical signs of infection. For analysis, only the strains isolated for the first time from a given patient were selected, in the situation of isolation of the same microbial species from the same case of infection or in screening, and recurrent strains were not included in further analyses. 

The active targeted screening by perirectal or rectal swab of patients was performed via the following:

- At admission to hospital, in case of suspected CRE colonization, e.g., antibiotic therapy, previous hospitalization, stay in the long-term-care facilities.
- Exposition or with close contact to patients with confirmed CRE infection during hospitalization.
- In an outbreak investigation, in the analysed period, there were 8 epidemics caused by CR *Klebsiella pneumoniae*, including 3 in Intensive Care Unit (ICU); most often (5 events, 62.5%) the cause was the KPC+ strain, followed by OXA-48.

**Figure 1:**
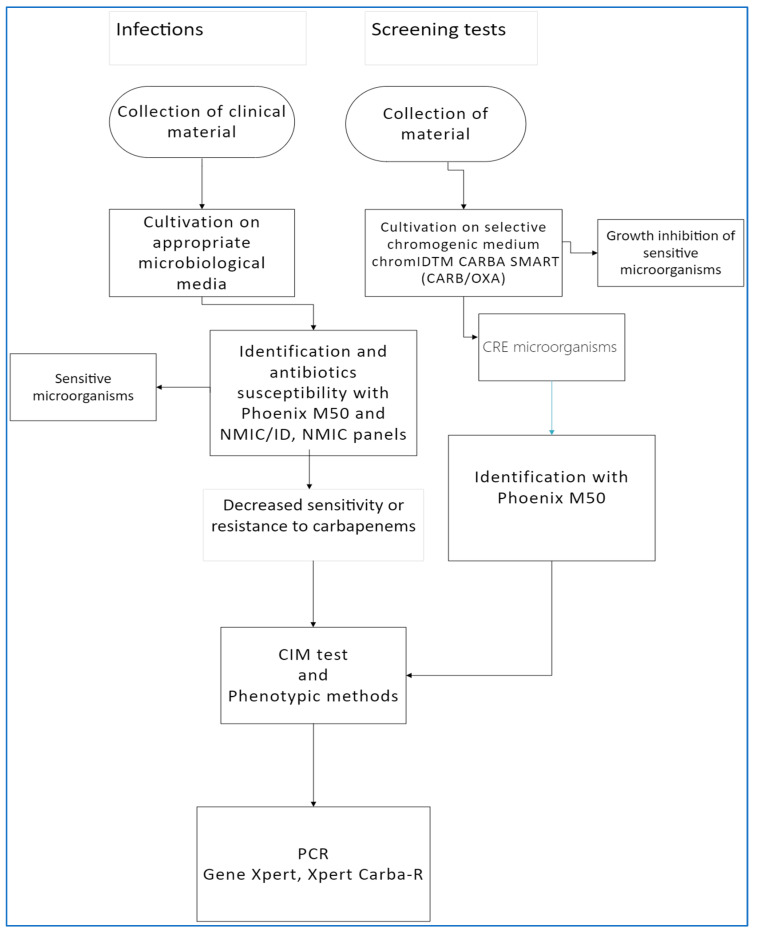
Diagram of CRE diagnostic procedure (CRE—carbapenem-resistant *Enterobacteriaceae*, CIM—Carbapenem Inactivation Method, and PCR—polymerase chain reaction).

Microbiological diagnostics was conducted at the Department of Microbiology of St. Barbara Specialized Regional Hospital No. 5 in Sosnowiec. Clinical materials were routinely inoculated onto individual sets of agar media depending on the type of material (Columbia agar +5% sheep blood, MacConkey, Chapman, Enterococcosel Agar, Pseudosel Agar, Sabouraud Agar), whereas to detect CRE in screening, a chromogenic medium was used chromID^TM^ CARBA SMART (CARB/OXA) (Biomerieux, Marcy-l’Etoile, France). (Figure 1). 

The isolated bacteria were identified using the Phoenix M50 automated system (Becton Dickinson, Warszawa, Poland). Antibiotic susceptibility testing was performed using the Phoenix M50 automated system, and combo panels were used for identification and drug susceptibility testing: NMIC/ID and NMIC extended panels (Becton Dickinson and Company, Sparks, MD, USA). Susceptibility tests were interpreted as recommended by EUCAST with the version for the years 2019–2021, respectively, i.e., 2019: 9.0, 2020: 10.0, 2021: 11.0 [11]. 

To diagnose isolates that are insensitive to carbapenem or with reduced sensitivity to ertapenem or meropenem, in order to confirm the presence of carbapenemase in the strain tested, the CIM (Carbapenem Inactivation Method) test [12] and phenotypic screening tests were used. CRE+ strains were confirmed with the molecular Xpert Carba-R test using the GeneXpert^®^ Instrument System (Cepheid, Sunnyvale, CA 94089 USA). Molecular tests for the presence of carbapenemase genes *bla*KPC, *bla*NDM, *bla*IMP, *bla*VIM, and *bla*OXA-48 were carried out in accordance with the manufacturer’s recommendations, from pure *Enterobacteriaceae* cultures grown on blood agar or MacConkey medium.

The study was conducted according to the guidelines of the Declaration of Helsinki and approved by Bioethical Committee of Sosnowiec Medical College in Sosnowiec (No. PW/WSM/36/17).

Statistical analyses were performed with the use of the PQStat statistical package, version 1.8.4. In the statistical analysis, relative and absolute frequencies were used for nominal variables. Chi2 test, Student’s t-test, and the Fisher exact test as appropriate were used to compare the groups. A test probability of *p* < 0.05 was considered significant. 

## 3. Results

In the study period, a total of 78,140 patients were hospitalized and 399 unique CRE isolates were detected, mainly from screening (n = 295, 74%) and from clinical material (n = 104, 26%) (Table 1). 

**Figure 2:**
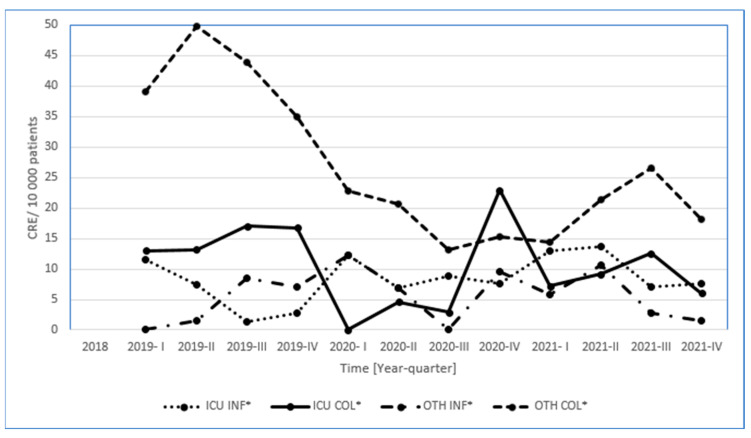
Incidence rate and colonization rate (CRE/10,000 patients) depending on the unit and year of hospitalization (CRE— carbapenem-resistant *Enterobacteriaceae*, ICU—Intensive Care Unit, INF—infection, COL—colonization, OTH—other units).

The incidence rate of CRE colonization was from 1.0 before the COVD-19 pandemic (first quarter of 2020) in Poland to 4.7 in the biggest wave of the pandemic (fourth quarter of 2020, Table 2), the incidence and colonization (CRE/10,000 patients) were also dependant on the type unit (Figure 2), and correlation analysis showed that increased screening is highly associated with an increase in the ratio of carriers to those with HAIs (R = 0.73, *p* = 0.004, Table 2). Thus, indirectly, the number of tests performed increases the frequency of carrier identification, but on the other hand, reduces the number of HAIs.

In 104 patients, symptomatic infection was found with an incidence rate of 13.3 per 10,000 hospitalizations. CR urinary tract infection (n = 29) with an incidence rate of 5.4 per 10,000 hospitalizations was found significantly more often than CR respiratory tract infection (n = 29) with an incidence rate of 3.7 (*p* < 0.001), CR bloodstream infection (n = 18) with an incidence rate of 2.3 (*p* = 0.01), and CR surgical site infection (n = 12) with an incidence rate of 1.5 (*p* = 0.035). In HAIs, *Klebsiella pneumoniae* (97 isolates) was most often isolated, with an incidence rate of 12.4 per 10 000 hospitalizations, followed by *Escherichia coli* (3 isolates), and *Serratia marcescens, Klebsiella oxytoca*, and *Klebsiella aerogenes* (1 isolate each, Table 3).

Genes mediating CRE were detected, in particular OXA-48 oxacillinase (71%), KPC carbapenemase (26%), New Delhi NDM metallo-β-lactamase (2.5%), and VIM (0.3%). Additionally, two isolates simultaneously had genes for two carbapenemases, i.e., OXA-48 and KPC (Table 4). The HAI *K. pneumoniae* OXA-48 was significantly more frequent than infections with *K. pneumoniae* KPC (*p* < 0.001) and NDM (*p* < 0.001) strains. Infections with *K. pneumoniae* OXA-48 were diagnosed more often than KPC (*p* = 0.027) (Table 4). 

All CRE strains were resistant to amoxicillin/clavulanic acid, ampicillin, cefepime, cefotaxime, ceftazidime, ceftriaxone, cefuroxime, ertapenem, and piperacillin/tazobactam. High resistance was also reported to imipenem, 84.5%, and meropenem, 68.2%. Resistance to amikacin was the lowest among aminoglycoside antibiotics and only amounted to 2%, and for the remaining aminoglycosides it was as follows: gentamicin (53.3%), netilmicin (96.5%), and tobramycin (59.3%). High resistance to antibiotics from the group of fluoroquinolones was also registered, i.e., 76.3%, except for the strain *S. marcescens*, which was 100% susceptible to these antibiotics. Resistance to aztreonam, colistin, and nitrofurantoin was as follows: 57.8, 61.2, and 25% (Table 5). Moreover, mixed resistance of CRE strains to trimethoprim-sulfamethoxazole was recorded in three species, i.e., *K. pneumoniae*, *K. oxytoca*, and *Proteus mirabilis* exhibited very high resistance to this antimicrobial agent, compared to other CRE strains. On the other hand, the lowest resistance out of all CRE strains was determined for ceftazidime in combination with avibactam. 

## 4. Discussion

In our study, there were 13.3 carbapenem resistance HAI cases per 10,000 hospitalizations, which is much higher than other parts of the world such as the 3.36–3.79 cases per 10,000 hospitalizations seen nationally in the United States before the COVID-19 pandemic [13]. Of the isolated bacteria, 74% was from screening material, and similar results obtained by Otter et al. [14].

An alarming phenomenon, which is worth giving attention to, is that more than 3/4 of the CRE strains were isolated through the screening of patients in our study, which indicates a very high degree of colonization. A high proportion of asymptomatic colonization with CRE strains is also confirmed by other Polish studies [15]. Hence, the key role in the prevention of the further spread of CRE strains is early detection of carriage in the patient through screening and implementation of contact precautions, in order to prevent the transmission of these strains in the hospital environment [8]. On the other hand, the prevalence of colonization was about 2%, the same as reported by Mathers et al. [16].

The targeted screening for identified CRE carriers in the studied hospital with or without weekly screening is the most cost-effective option to limit the spread of CRE [17]. However, despite the planned actions, the morbidity associated with CR *Enterobacteriaceae* was very high, more than three times higher than the expected value (USA, before the COVID-19 pandemic [13]). At present, good practise would involve screening all patients admitted to the hospital during treatment and at discharge, which would allow assessing the patient’s exposure to colonization or infection with CRE strains and therefore verifying the tightness of the implemented procedural and decision-making standards. Unfortunately, similarly high values are now reported around the world, which are linked by researchers to the impact of the COVID-19 pandemic and the high consumption of antibiotics associated with it.

In 2020, carbapenem resistance was generally lower in the north and west of the WHO European Region, where 16 out of 41 (39%) countries/areas reported a percentage of resistance below 1% and the next quarter of EU/EEA countries reported CR *K. pneumoniae* above 10%. However, 6 (15% out of 41 countries/areas) reported a 50% resistance, while in our study it was 30% [18]. 

The phenomenon of increasing resistance to carbapenems in *K. pneumoniae* observed in Poland from 2016 was associated with the spread of KPC, especially the NDM-type carbapenemase. In Poland, the proportion of resistance of *K. pneumoniae* strains to carbapenems has increased in recent years from 0.5% in 2015 to 8.2% in 2020 [18]. 

At present, in Europe and in the world, the problem of the acquisition and spread of carbapenem resistance with respect to microbial drug resistance takes a special place. The greatest epidemiological and clinical significance in this phenomenon is occupied by *Enterobacterales* which produce carbapenems of the type OXA, KPC, and NDM. The occurrence of CRE is associated with the probability of developing resistance in these strains to all available antibiotics. Furthermore, carbapenemase genes are located on mobile genetic elements, which can easily spread between strains of the same or different species by gene transfer [19]. Data from the EARS-Net network for 2020 demonstrate that the determinants of carbapenem resistance were more frequently reported for the strains of *K. pneumoniae* than the strains of *E. coli*. 

A greater part of the identification of *K. pneumonaie* NDM compared to OXA-48 was confirmed in other studies [20,21]. However, our own analysis of the tested CRE strains demonstrated a much higher proportion of *K. pneumoniae* strains with OXA-48 carbapenemase than NDM carbapenemase. A greater number of the recorded *K. pneumoniae* OXA-48 strains was associated with the occurrence of periodic epidemic outbreaks in hospital units, stemming from the transmission of *K. pneumoniae* OXA-48+ under hospital conditions. The main route of transmission of CRE is the contact route in healthcare settings, however, NDM and OXA carbapenemases can also spread in community environments [22]. It is important to emphasize the significance not only of the hospital screening but also appropriate infection prevention and control practises, including proper hand hygiene, to prevent the spread of carbapenem-resistant strains [23].Unfortunately, a great role in the transmission of CRE strains is played by healthcare professionals who, on the one hand, are aware of the importance of infection prevention and control measures, such as hand hygiene, in preventing the transmission of CRE, but on the other hand, have identified barriers in this regard, such as time constraints [24]. In previous studies, the authors drew attention to the large gaps in knowledge and skills concerning hand hygiene among Polish healthcare workers [25].

CRE strains are not only a clinical and epidemiological problem, due to the possibility of global spread and gene transfer, but also due to high resistance to commonly used antibiotics [8,26,27]. The tested isolates exhibited sensitivity to gentamicin, amikacin, and ceftazidime-avibactam, but a significant number of them were extensively drug resistant, XDR, which was also observed in other studies [28]. 

Very limited sensitivity of XDR *Klebsiella pneumoniae* strains, sensitivity mainly regarding aminoglycosides, was also described by Kim et al. [26], which significantly restricts therapeutic options [29,30]. The treatment of infections caused by CRE consists in the application of aminoglycosides, fosfomycin, polymyxin, and tigecycline in monotherapy or the use of combination therapy, including high-dose tigecycline, prolonged infusion of high-dose carbapenems, and dual carbapenem therapy. High doses of antibiotics, including carbapenems, correlate with better outcomes in the treatment of infections caused by CRE strains with a carbapenem MIC <8 mg/L. Moreover, the effectiveness of treatment can be boosted owing to the newly available antibiotics: ceftazidime/avibactam, active against the KPC and OXA-48 producers, and meropenem/vaborbactam, which works against KPC producers [29,30,31]. 

## 5. Strengths and Limitations

As factors increasing the value of the study, the analysis of a large group of patients in the study period should be taken into account, which translates into the analysis of a large number of screening tests.

The limitation of this study was the lack of available characteristics of all patients due to a huge population and the lack of access to the entire database of patients admitted in the analysed period. The retrospective nature of the study also does not allow for the surveillance and evaluation of the insulation measures implemented in the audited unit, which can affect the higher ratio of carriers to identified nosocomial infections.

## 6. Conclusions

The demonstrated data confirm the urgent need for continuous monitoring of CRE strains, as well as controlling the implementation of procedures to prevent the transmission of CRE strains by medical as well as support personnel. Optimization of infection prevention including hand hygiene and transmission-based precautions and antibiotic stewardship is an important factor in reducing the occurrence of CRE infections. 

## Figures and Tables

**Table 1:** Distribution of clinical specimens of CRE.

Specimen	No. CRE				Incidence Rate Per 10,000 Hospitalizations
	2019	2020	2021	Total (%)	
Respiratory specimens	0	12	17	29 (7)	3.7
Urine	16	11	15	42 (11)	5.4
Skin and soft tissue infections	4	5	3	12 (3)	1.5
Blood	8	3	7	18 (5)	2.3
Subtotal	28	33	43	104 (26)	13.3
Screening swabs	159	57	79	295 (74)	
Total	187	88	121	399 (100)	

CRE—carbapenem-resistant *Enterobacteriaceae.*

**Table 2:** Characteristics of screening tests and results of it.

Quarter	Year	No. Admission	No. Screening Tests	Screening Tests (%)	Results CRE+	Results CRE+ (%)	CRE-HAI	Incidence CRE-HAI *	CRE+/CRE-HAI **
I	2019	6914	1877	27.1	36	1.9	8	11.6	4.5
II	2019	6817	1951	28.6	43	2.2	6	8.8	7.2
III	2019	7050	1889	26.8	43	2.3	7	9.9	6.1
IV	2019	7151	1615	22.6	37	2.3	7	9.8	5.3
I	2020	6551	1433	21.9	15	1.0	10	15.3	1.5
II	2020	4366	712	16.3	11	1.5	6	13.7	1.8
III	2020	6804	1143	16.8	11	1.0	6	8.8	1.8
IV	2020	5237	423	8.1	20	4.7	9	17.2	2.2
I	2021	6914	660	9.5	15	2.3	13	18.8	1.2
II	2021	6571	906	13.8	20	2.2	16	24.3	1.3
III	2021	7137	1165	16.3	28	2.4	7	9.8	4.0
IV	2021	6628	940	14.2	16	1.7	6	9.1	2.7

CRE—carbapenem-resistant *Enterobacteriaceae;* HAI—healthcare-associated infection; * per 10,000 pds; ** $\frac{No.\;results\;CRE+}{No.\;CRE-HAI}.$

**Table 3:** Species distribution among CRE from HAI.

Species	No. CRE				Incidence Rate Per 10,000 Hospitalizations
	2019	2020	2021	Total (%)	
*Klebsiella pneumoniae*	25	32	40	97 (93)	12.4
*Klebsiella oxytoca*	0	0	1	1 (1)	0.1
*Klebsiella aerogenes*	0	0	1	1 (1)	0.1
*Escherichia coli*	3	0	0	3 (3)	0.4
*Serratia marcesces*	0	1	0	1 (1)	0.1
*Proteus mirabilis*	0	0	1	1 (1)	0.1
Total	28	33	42	104 (100)	13.3

CRE—carbapenem-resistant *Enterobacteriaceae;* HAI—healthcare-associated infection.

**Table 4:** Distribution of carbapenemase genotypes by species.

	Isolated Strains N			
	Screening	HAI	Total	Prevalence (%)
*Klebsiella* spp. *n* = 393				
OXA-48	215	64	279	76.6
NDM	4	6	10	3.3
KPC	75	27	102	28.8
OXA-48, KPC	0	2	2	1.0
*Escherichia coli n* = 4				
OXA-48	1	3	4	100.0
NDM	0	0	0	0.0
KPC	0	0	0	0.0
OXA-48, KPC	0	0	0	0.0
*Proteus mirabilis n* = 1				
OXA-48	0	0	0	0.0
NDM	0	0	0	0.0
KPC	0	0	0	0.0
OXA-48, KPC	0	0	0	0.0
VIM	0	1	1	1.0
*Serratia marcescens* *n* = 1				
OXA-48	0	1	1	100.0
NDM	0	0	0	0
KPC	0	0	0	0
OXA-48, KPC	0	0	0	0
VIM	0	0	0	0

CRE—carbapenem-resistant *Enterobacteriaceae;* HAI—healthcare-associated infection; KPC—*Klebsiella pneumoniae* carbapenemase; New Delhi NDM metallo-β-lactamase; oxacillinase OXA-48; VIM—Verona integron-encoded metallo-β-lactamase.

**Table 5:** Antimicrobial resistance of common CRE—carbapenem-resistant *Enterobacteriaceae*, only bacilli isolated from healthcare-associated infections.

Antibiotics	*Klebsiella Pneumoniae*			Other *	Total
	2019	2020	2021	2019–2021	2019–2021
	(*n* = 25) (%)	(*n* = 30) (%)	(*n* = 39) (%)	(*n* = 7) (%)	(*n* = 104) (%)
β-Lactam Antibacterials, Penicillins					
ampicillin	100	100	100	100	100
amoxicillin + clavulanic acid	100	100	100	100	100
piperacillin + tazobactam	100	100	100	100	100
Cephalosporins					
cefuroxime	100	100	100	100	100
cefotaxime	100	100	100	100	100
ceftazidime	100	100	100	100	100
ceftriaxone	100	100	100	100	100
cefepime	100	100	100	100	100
Cephalosporin+ Non-β-Lactam β-Lactamase Inhibitor					
ceftazidime-avibactam	2	8	6	0	0.7
Carbapenems					
IPM	65	91	89	85	84.5
MEM	63	75	67	68	68.2
ETP	100	100	100	100	100
Aminoglycosides					
amikacin	8	8	11	0	2
gentamicin	88	82	40	50	53.3
tobramycin	98	98	93	52	59.3
netilmicin	87	NT	NT	100 ***	96.5
Fluoroquinolones					
ciprofloxacin	100	100	95	72	76.3
levofloxacin	100	100	95	72	76.3
Other Antibacterials					
aztreonam	100	95	95	44.3 **	57.8
colistin	75	92	73	60	61.2
fosfomycin	62	63	33	NT	55
nitrofurantoin	NT	NT	NT	25 ***	25
trimethoprim-sulfamethoxazole	98	92	96	40	49.5

n—Total isolates, NT—not tested. * *K. oxytoca*, *K. aerogenes*, *E.coli*, *S. marcxescens*, and *P. mirabilis*. ** Tested only for *K. oxytoca*, *E.coli*, and *Serratia marcescens*. *** Tested only for *E. coli*.

## Data Availability

The datasets generated or analysed during this study are available and can be obtained, at request, from Iwona Pawłowska (e-mail: ivi5@op.pl) on reasonable enquiry.
